# How to Produce, Identify, and Motivate Robust Psychological Science: A Roadmap and a Response to Vize et al.

**DOI:** 10.1177/10731911241299723

**Published:** 2024-11-29

**Authors:** E. David Klonsky

**Affiliations:** 1 The University of British Columbia, Vancouver, British Columbia, Canada

**Keywords:** metascience, preregistration badges, robust science, psychological science, open science, replication crisis, transparency

## Abstract

Some wish to mandate preregistration as a response to the replication crisis, while I and others caution that such mandates inadvertently cause harm and distract from more critical reforms. In this article, after briefly critiquing a recently published defense of preregistration mandates, I propose a three-part vision for cultivating a robust and cumulative psychological science. First, we must know how to produce robust rather than fragile findings. Key ingredients include sufficient sample sizes, valid measurement, and honesty/transparency. Second, we must know how to identify robust (and non-robust) findings. To this end, I reframe robustness checks broadly into four types: across analytic decisions, across measures, across samples, and across investigative teams. Third, we must be motivated to produce and care about robust science. This aim requires marshaling sociocultural forces to support, reward, and celebrate the production of robust findings, just as we once rewarded flashy but fragile findings. Critically, these sociocultural reinforcements must be tied as closely as possible to rigor and robustness themselves—rather than cosmetic indicators of rigor and robustness, as we have done in the past.

Klonsky (2024) argues that Campbell’s Law explains the replication crisis, and that preregistration badges are history repeating. In brief, psychological science has repeatedly harmed itself by converting useful scientific tools—such as hypotheses, *p*-value thresholds, and multi-study designs—into cosmetic indicators of scientific rigor and goals in and of themselves. The result has been distorted use of these tools (e.g., hypothesizing after results are known, *p*-hacking, misuse of researcher degrees of freedom), and decades of findings that do not replicate. Klonsky (2024) further argues that preregistration mandates are history repeating: once again a useful tool has been converted into an indicator of scientific rigor, and a goal in and of itself—even a literal badge to be obtained and displayed. Indeed, there is already evidence that preregistration mandates encourage the presence of preregistrations without the accompanying rigor (Bak-Coleman & Devezer, 2024; Claesen et al., 2021; van den Akker et al., 2023). Klonsky (2024) concludes with an alternate vision in which multiple features are understood to support robust science (including large sample sizes, valid measures, robustness checks, and preregistration), none are canonized, and sociocultural forces are marshaled to encourage robust science, just as they once encouraged flashy but fragile science.

## The Response by Vize et al.

Vize et al. (2024) issue a thoughtful and passionate response to this perspective. They feel that Klonsky (2024) underappreciates the purpose and benefits of preregistration as an antidote to the replication crisis and presents an unconvincing alternative vision. It is important for their perspective to be heard. I greatly respect the authors as researchers and thinkers. Solving the replication crisis requires full-throated discussion and wide participation across the field.

At the same time, I am comfortable standing by my original thesis: the important points raised by Vize et al. either do not directly engage with my core arguments or were anticipated and addressed in my initial article. I give four examples. First, Vize et al. mistakenly felt I was criticizing “preregistration itself,” when in fact, I was critiquing preregistration as a mandate. For example, Klonsky (2024) repeatedly praises preregistration itself, *never* criticizes preregistration itself, and consistently distinguishes the benefits of a tool itself from concerns about the tool becoming a mandate.^1^ Second, the first “misunderstanding” Vize et al. address is the idea that preregistration cannot accommodate robustness checks—but Klonsky (2024) is clear that preregistration *can* accommodate robustness checks when done well, and Vize et al. correctly cite this part of my manuscript (although they sidestep my suggestion that rewarding only preregistration, and not robustness checks, will sideline robustness checks). Third, Vize et al. state that I view “increasing [statistical] power” as a benefit of preregistration. In contrast, Klonsky (2024) does not say this or make any claim about the impact of preregistration on power. As a final example, Klonsky (2024) takes for granted that undisclosed researcher degrees of freedom are harmful because they mislead about the stringency of the tests reported. Vize et al. felt I should have made this connection explicit; in fact, they make this a central point in their reply. To be clear, I agree about these benefits of transparency (even though there are questions about how Vize et al. links preregistration to the concept of test “severity”; see Rubin, 2024). However, as a reminder, Klonsky (2024) was about Campbell’s Law and its damaging implications when useful tools like hypotheses, *p*-value thresholds, and preregistration become mandated. In the spirit of efficiency, I will resist the urge to further recapitulate the detailed arguments in Klonsky (2024) as a response to Vize et al. If you are intrigued by my thesis, please see Klonsky (2024).

Vize et al. also raise broader considerations I am eager to address. These include the role of preregistration in relation to other approaches that can help solve the replication crisis, as well as the role of sociocultural forces in this process. While Vize et al. offer thoughtful considerations, their emphasis on preregistration may inadvertently downplay the critical and necessary factors to solve the replication crisis and cultivate a robust and cumulative psychological science. Building on this discussion, I now present a three-part roadmap for how our field can produce, identify, and motivate robust science.

## A Roadmap for Robust Psychological Science

A healthy science is a cumulative one: one in which robust findings are accumulated to provide a foundation for subsequent work and progress (Curran, 2009). In recent decades, psychological science has fallen short of this ideal (Gendreau, 2002; Open Science Collaboration [OSC], 2015; Sharpe & Goghari, 2020; Simmons et al., 2011). We are therefore left with a question of the highest importance: How do we become a robust and cumulative science?

I answer this question in three parts: (a) How to produce findings that are likely to be robust, (b) How to determine which findings are (and are not) robust, and (c) How to ensure our field is motivated to produce and value robust findings, rather than the flashy, fragile findings prioritized in the past. My answers expand on ideas introduced in Klonsky (2024), while challenging some ideas and potential misperceptions in Vize et al., with the goal of advancing discussion on these key issues.

### How to Produce Findings Likely to Be Robust

To produce robust findings, we must have a shared understanding of factors likely to generate such findings. These include appropriate sample sizes, valid measures, and honest/transparent scientific practice. I address each of these below.

#### Sample Size

It would be ideal if researchers could obtain data from every member of a target population. Since doing so is usually unrealistic, we instead study samples from a population, with the hope that patterns observed in the sample reasonably approximate true patterns in the larger population. Of course, effects observed in a sample are imperfect estimates of the population effects. Inferential statistics—such as confidence intervals—exist to characterize this gap as precisely as possible. Narrow confidence intervals suggest higher confidence that a sample effect is reasonably similar to the population effect, whereas wide confidence intervals suggest uncertainty.

Critically, the size of the sample we study directly impacts the size of the confidence interval around our effect-size estimate.^2^ Because larger samples are less susceptible to sampling error, they better represent the population from which they are drawn, and thereby permit more accurate approximations of population effects. Conversely, small samples are highly susceptible to sampling error and provide very uncertain estimates of population effects. In fact, studies with small-sample sizes frequently yield effect sizes that are way off the mark—including large effect sizes when the population effect is negligible, and negligible effect sizes when the population effect size is large.

For whatever reason, psychological scientists do not act like this fact is true. We routinely choose sample sizes that are far too small, while offering conclusions about which effects are “significant” or null (Liu et al., 2023; Szucs & Ioannidis, 2017). This practice introduces a ton of random error into the effect sizes that are published, as well as systematic error since journals are more likely to publish inaccurate effect-size estimates that happen to be large (Kuhberger et al., 2014). It is an inescapable reality: small-sample sizes lead to fragile findings. This is one reason why projects pooling resources to create large sample sizes often fail to replicate highly cited effects from small-sample studies (Klein et al., 2022; OSC, 2015; Poldrack et al., 2017).

Even if scientists struggle to understand the formal mathematical relationship between sample size and confidence intervals, we can understand the relationship intuitively. For example, we expect polls of political elections to include several hundred or thousand people. If a poll included just 60 people, we would not take it seriously. We would understand that the results might be way off, and that a second poll of the same sample size could yield a very different result. Moreover, if we cared about understanding a population’s perceptions of political candidates, we would seek a larger poll, *not* the *p*-value addressing whether the difference between candidates’ support in the small poll was “significant.” In other words, we would take for granted that a political finding based on a small-sample size is likely to be fragile.

We must bring this intuition into our psychological science. We should not have more confidence in a psychological study of 60 people, or even 100 people, than we would about a political poll of the same sample size. In trying to create a more robust science, it is imperative that the field—including researchers, funding agencies, and journal editors—prioritize larger sample sizes (Liu et al., 2023; Szucs & Ioannidis, 2017).

#### Measurement Validity

While sample size is crucial for reducing sampling error, robust findings also depend on valid measurement. Without valid tools to assess psychological constructs, even large samples can produce unreliable results. This brings us to the next key factor: measurement validity.

It has long been understood that producing psychological knowledge requires the valid measurement of psychological constructs (Cronbach & Meehl, 1955). To a large extent, the field of psychological science understands this. Entire journals—such as *Assessment* and *Psychological Assessment*—are devoted to studying the reliability, validity, and utility of such measures, and articles about constructing valid measures have thousands of citations (Clark & Watson, 1995).

At the same time, there are large portions of psychological science that consistently ignore basic principles of measurement. For example, many biological (Elliot et al., 2020; Moriarity & Alloy, 2021) and behavioral measures (Parsons et al., 2019; Xu et al., 2024) have been used for decades despite either no or poor evidence for their reliability. There is no validity without reliability. Predictably, neglecting measurement reliability and validity has produced decades of fragile science, and numerous findings in these fields that fail to replicate (Border et al., 2019; Chapman et al., 2019; Masouleh et al., 2019).

Studies neglecting measurement basics not only pollute the field with false findings but also waste countless hours of effort and millions of dollars in grant funding. It is essential we learn from these mistakes. Valid measurement is necessary for the cultivation of a robust and cumulative psychological science (Lilienfeld & Strother, 2020).

#### Honesty/Transparency

A major contribution to the replication crisis has been a lack of transparency, or even honesty, about the researcher degrees of freedom used to achieve the findings we publish (Simmons et al., 2011). Researchers have cherry-picked findings that achieve desired *p*-values, formulated explanations for them post hoc, and presented the explanations as hypotheses and the analyses as if they had been planned on the basis of the fake hypotheses (Kerr, 1998). Practices such as *p*-hacking, selective reporting, and publication bias have resulted in decades of false-positive findings (Friese & Frankenbach, 2020; Masicampo & Lalande, 2012).

We must appreciate why a lack of honesty and transparency leads to the publication of fragile findings. The size of effects we observe in our samples are driven by two factors: signal and noise. If enough analyses are conducted of negligible population effects, noise alone ensures that some will yield effects that appear large or meet traditional thresholds of statistical significance. Therefore, an effect yielded by a focused analytic plan is not equal to an effect yielded by cherry-picking from among numerous analyses. The latter is far less likely to be robust. To dishonestly and non-transparently publish cherry-picked findings is to pollute the literature with fragile findings disguised as robust ones. We must come to view this habit as scientific malpractice (a similar point was subsequently articulated in Miller et al., in press).

Honesty and transparency do not mean avoiding exploratory analyses. Rather, we simply must be honest about the analyses that were done so that findings can be properly contextualized. At its core, honesty and transparency are a choice: we can choose to make them priorities, such that the information in our Methods and Results sections is accurate and thorough. At the same time, honesty and transparency can be difficult. There may be pressures to present less robust findings in a manner that maximizes their novelty, interest, and publishability, while concealing their fragility. It is also possible to simply forget, or misremember, the history of analyses that have been conducted from start to finish. Honestly and transparency require commitment, effort, and care.

Some wish to mandate preregistration as a solution for achieving transparency (Vize et al., 2024). However, I think this is a mistake. First, it is quite possible to be non-transparent, or dishonest, in preregistrations. If we can be dishonest for decades about the hypotheses we publish (Kerr, 1998), we can be dishonest about the preregistrations we submit (e.g., preregistering after results are known; Yamada, 2018; see Klonsky, 2024 for elaboration). Peer reviewers could evaluate the quality of the preregistration as written and evaluate the article in the context of this preregistration, but would not know if the preregistration was transparent and honest. It also would not be transparent if researchers disproportionately submit some preregistered studies/analyses for publication (e.g., those producing the most interesting or “significant” findings) but not others (e.g., those with less interesting or null findings). Realistically, it is impossible to audit all preregistrations for accuracy and transparency. It would therefore be wrong to assume that preregistration equals transparency.

Second, even if we preregister accurately and honestly, we must still transparently and honestly disclose in the article the unplanned analyses we conducted, rather than reporting some but concealing others. In fact, even Vize et al. suggest that unplanned analyses can and should be presented transparently and honestly in published articles. If unplanned analyses can be presented transparently and honestly in articles, so can preplanned analyses.

I therefore disagree with Vize et al. that a preregistration norm is necessary for creating transparency. In the end, regardless of whether or not preregistration is used, we are trusting authors to be transparent and honest in the published articles themselves. I fully agree that preregistration can be a valuable tool to help authors keep and share records of planned analyses, among other benefits. But it is not more than a tool. There are other approaches for keeping good records and accomplishing transparency and honesty—and, in fact, such approaches are *always* necessary give that preregistration only addresses a part of the analytic enterprise. The key is not the specific tools used, but that we learn to report relevant methodological information fully, transparently, and honestly in the articles we publish.

In fact, I believe that Transparency should be a standard part of scientific articles. For example, in a non-preregistered paper I am currently working on with a student, we conducted confirmatory factor analyses (CFA) on two large item sets. We used a maximum likelihood (ML) estimation procedure and were surprised to find a sub-optimal fit for one of the item sets. We then realized that the nature and distributions of the items were a poor fit for ML, and based on a check of the CFA literature, re-ran the analysis using diagonally weighted least squares (DWLS), which resulted in a better fit. In the Results section we are drafting, we report results for both estimation methods (ML and DWLS); in a Transparency subsection, we document that ML was our original plan, and that DWLS was a researcher degree of freedom we only pursued after seeing the ML results. This approach may be similar to the Research Transparency Statement now required by the journal *Psychological Science* (Hardwicke & Vazire, 2024). Such a section not only provides opportunities for authors to more carefully consider and report the history of analytic decisions, but also encourages reviewers to ask questions and request additional information to improve transparency, and gives important context that allows readers to better interpret the findings. Importantly, this also keeps the focus on transparency itself in the articles we publish, rather than if a particular tool has been used.

That said, there are some studies for which I believe independently monitored and audited preregistrations are essential to ensure honesty and transparency: high-stakes work in which the investigator is biased toward a certain outcome. Examples include studies of clinical tools or treatments in which investigators have intellectual or financial stakes in the tool’s/treatment’s effectiveness, or replication studies of highly cited effects to which an investigator’s reputation is tied. Human biases are powerful even for well-meaning scientists, and some cases require an enforced “straitjacket” (Klonsky, 2024) against the abuse of researcher degrees of freedom.

In sum, there are many paths to honesty and transparency. Different situations can call for different approaches. While some characterize preregistration as essential for achieving transparency (Vize et al., 2024), I view preregistration as a relevant tool that is neither necessary nor sufficient: you can have honesty and transparency without preregistration, and you can have preregistration without honesty or transparency. The key is that honesty and transparency are a choice we must make if we are to create a robust and cumulative psychological science.

#### Badges for Sample size, Measurement, and Honesty/Transparency?

Although I have argued for the importance of sample size, measurement, and honesty/transparency in generating robust findings, it is important that we not canonize or award badges to purported indicators of these ideals. First, per Campbell’s Law, doing so would corrupt their use for reasons detailed in Klonsky (2024). Second, there are always thoughtful, defensible, and important exceptions that should not be discouraged. Badges or other forms of rewards or mandates can discourage important research that does not conform to the reward or mandate. For example, experimental case designs, pilot/feasibility studies, and the accumulation of small-sample studies for hard-to-recruit populations (e.g., Autism, infants) are important and useful and may be discouraged if sample size mandates/badges/thresholds make them harder to publish or less likely to be viewed favorably. Similarly, important psychological phenomena sometimes require provisional or novel assessment when no valid measures exist (measurement exception), and transparency/honesty can be achieved in many ways (preregistration badge exception). Thus, even when design features and tools are useful, we should not elevate them to badges or mandates as doing so—perhaps counterintuitively—causes more harm than good (Klonsky, 2024).

### How to Verify Which Findings Are (and Are Not) Robust

So far, I have emphasized features likely to produce robust findings. However, even with sufficient sample sizes, valid measures, and strong honesty/transparency, non-robust findings can still emerge, although less likely. In addition, there can be ambiguity about what constitutes sufficient sample sizes, appropriate measures, and adequate transparency. In short, while these practices increase the chances of producing and publishing robust findings, they are not a guarantee. Therefore, it is essential that we understand as a field how to identify robust (and non-robust) findings. In contrast to Vize et al., my vision puts robustness checks at the center of this enterprise. In this section, I argue that we should trust findings are robust to the extent that they replicate across reasonable alternative analyses, measures, samples, and investigative teams. I describe these below.

#### Analytic Robustness

There are almost always numerous reasonable approaches to analyzing data in service of a given research question (Gelman & Loken, 2013). For example, analyses can be done with or without the exclusion of outliers, with or without transformations of non-normal variables, or with or without covariates that may be viewed as influencing or confounding the effect of interest, among many other possible choices. One way to increase confidence in the robustness of a given finding is to verify that the finding persists when conducting other reasonable variations of the analysis. Alternatively, if the finding changes meaningfully or disappears in other reasonable versions of the key analysis, confidence in the finding’s robustness should decrease. Thus, analytic robustness checks should be considered an indispensable part of scientific practice (Klonsky, 2024; Nuijten, 2022; Steegen et al., 2016).

In fact, I would suggest that Robustness Checks should be a standard part of Results sections. This is a practice we are beginning in my lab. For example, in the research I previously mentioned conducting with a student, we formed scales based on a series of factor analyses. One of the analyses suggested an item might not belong to a given scale, but other analyses suggested the item did belong. We opted to include the item in the scale for the subsequent analyses in which we correlate it and other scales to another variable. However, for our Robustness Checks section, we are rerunning the correlations and partial correlations with the item omitted from the scale to see if there is meaningful impact on the findings. We also noticed that some of our variables violate assumptions of normality, so we are rerunning the correlational analyses using a transformation to achieve a more normal distribution, as well as a non-parametric approach (Spearman) which is less sensitive to outliers. These analyses will help us determine whether our main findings are robust across reasonable alternative analytic approaches.

Notably, some analytic robustness checks only become apparent after engaging with the data and viewing results. This is not a problem scientifically. Analytic robustness checks planned a priori are *not* inherently more valuable than post hoc robustness checks. In fact, the most valuable analytic robustness checks might be conceived by authors while writing up results, by reviewers during the editorial process, or by others postpublication. Analytic robustness should be on our minds throughout the research enterprise.

#### Measurement Robustness

Validated measures help reduce the chances of spurious effects because their scores reflect more signal than noise. However, even validated measures contain enough noise to sometimes cause spurious effects. Thus, when possible, it is useful to determine whether a finding of interest persists across conceptually equivalent but different measures of the same construct. For example, as described in Klonsky (2024), I once found a relationship between a particular psychophysiological index and a measure of borderline personality disorder symptoms. However, this finding then failed two robustness checks, which reduced my confidence in the finding and led me not to publish it. The first was an analytic robustness check: the finding disappeared after removing an outlier and rerunning the same analysis. The second was a measurement robustness check: the finding disappeared when using a second measure of borderline personality disorder that happened to be included in the data set. As a result, even though my preplanned analysis yielded a *p* < .05 result, I decided the finding was not robust.

Measurement robustness checks will not be as pervasive as analytic robustness checks, since most studies do not contain multiple measures of the same construct. That said, it is still a useful type of robustness check to keep in mind, and plan for, when applicable. For example, research programs or papers that include replication studies can integrate this concept into the follow-up by adding an additional measure of the key construct(s).

To be clear, measurement robustness does not refer to whether substantively different measures of a construct yield different findings. Some measures of a given construct are not conceptually equivalent. For example, if a variable of interest correlated with a measure of anxiety emphasizing physiological symptoms, but not with a measure of anxiety emphasizing subjective worry, it would be unclear whether to consider this an example of failed robustness or a case in which the variable genuinely has different relationships to physiological versus subjective symptoms of anxiety. Further research would be needed.

#### Sampling Robustness Checks

A third type of robustness check is whether a given finding repeats when the same study is conducted in a different sample. “Same study” means the same analyses, same measures, and same sampling procedures from the same population—but a second sample. While this type of robustness check may seem synonymous with how some use the term “replication,” I suggest it is more specific. In a sampling robustness check, the second sample is theoretically identical to, and interchangeable with, the first. Thus, this kind of robustness is meant to address the potential role of statistical sampling variability on the original finding.

In contrast, the term “replication” can be used more broadly and less precisely. For example, someone may ask whether a given finding would replicate in an older sample. In this case, the replication would *not* be a robustness check addressing sampling variability. Instead, this question addresses whether a given finding might apply to a different population—which is a question about generalizability not robustness.

#### Robustness Across Investigative Teams

Finally, it is important to know whether a given finding persists when the same study design is applied across investigative teams. Human biases are powerful. Despite our best efforts, human biases can systematically impact findings detected or reported in a given study (Nickerson, 1998; Simmons et al., 2011; Wilholt, 2009). Thus, when a finding is replicated by the same investigative team, it is unclear if the finding replicated because it is robust or because it is consistently produced by an undetected or undisclosed systematic bias. Ideally, we would find unbiased investigators to test the effect. However, all people have biases, so when it comes to independent replication, the best we can hope for is different investigative teams with presumably different biases. Findings that replicate across different investigative teams are more likely to be robust, and findings that fail this check are less likely to be robust (OSC, 2015).

### How to Motivate the Production and Identification of Robust Findings

So far I have addressed (a) How to produce robust findings and (b) How to identify robust findings. However, even if this knowledge is readily available, researchers must be motivated to use it. This is no small challenge. Historically, psychological researchers and journals have been disproportionately motivated to publish and promote novel but fragile findings. Antonakis (2017) refers to this pathology as “neophilia.” In the words of Simine Vazire, editor of the flagship journal *Psychological Science*, researchers and journals prioritized “hype” over “rigor” (Vazire, 2024). In my own words, shared with the Association for Psychological Science in 2014

Journals . . . are littered with underpowered studies, *p* < .05 fishing expeditions, and clever or “sexy” conclusions that are not justified by the data—these studies get published as long as they conform to aesthetic norms and yield clever and/or attention-getting headlines. (Association for Psychological Science [APS], 2014)

In short, for decades, our field’s culture of reward and punishment has motivated flashy, fragile work—not robust work.

How do we motivate researchers differently? The answer is simple if challenging to achieve: we must abandon the old culture and replace it with one that rewards robust research and punishes fragile work.

As I note in Klonsky (2024), this culture change is underway. This include numerous efforts to distinguish robust and non-robust findings (e.g., APS, 2023; OSC, 2015; datacolada.org; replicats.research.unimelb.edu.au), the development of organizations devoted to the improvement of scientific practice such as the Center for Open Science (https://www.cos.io) and the Society for the Improvement of Psychological Science (https://improvingpsych.org), and journal editorial visions and policies that prioritize methodological rigor over “significant” findings (Vazire, 2024). I am also heartened there is growing recognition that preregistration badges, although well-intentioned, may “mislead more than they inform” (Hardwicke & Vazire, 2024) and “harm rather than safeguard psychological science” (Klonsky, 2024).

In creating a culture that rewards practices likely to lead to robust work and punishes practices likely to lead to fragile work, I believe we must be supremely thoughtful and careful about the rewards and punishments we put in place. Historically, in trying to reward robust science, we have instead rewarded features that came to falsely signify robust science, such as the presence of hypotheses, *p*-values below .05, and multi-study designs (Klonsky, 2024). In my view, preregistration mandates are a similar error: “preregistration mandates are positioned as a way to reinforce strong science, but in reality they reinforce achieving badges” (Klonsky, 2024).

Vize et al. suggest I am reserving my skepticism, or “cynicism,” for preregistration mandates, but not the sociocultural forces I suggest can successfully reinforce robust science. I disagree! For better or worse, cynicism is at the core of how I view the replication crisis and its solutions. I cynically believe that reward and punishment are the most important motivators of scientist behavior, not good intentions. I also cynically believe that canonizing certain tools—like hypotheses, *p*-value thresholds, multi-study designs, and now preregistration—encourages the presence of exactly those tools, not the robust science they are thought to create (Klonsky, 2024).

The lesson is this: our sociocultural rewards and punishments must be tied as closely as possible to the production and identification of robust science. The expanded efforts to identify and publicize fragile findings (APS, 2023; OSC, 2015; datacolada.org; replicats.research.unimelb.edu.au) are a good example because they create a powerful incentive not to produce fragile work. In the words of the eminent physicist Richard Feynman (1974), scientists must learn that:

the truth will come out. Other experimenters will repeat your experiment and find out whether you were wrong or right . . . although you may gain some temporary fame and excitement, you will not gain a good reputation as a scientist if you haven’t tried to be very careful in this kind of work.

A second example is the development of journal editorial practices that favor manuscripts high on “rigor” and “humility,” and that regard “hype” and “unwarranted bold claims” as a basis for rejection (Vazire, 2024). These developments move beyond past trends of rewarding particular tools or design features (Klonsky, 2024), and incentivize rigor and robustness themselves. This is the kind of change we need.

A comprehensive account of sociocultural rewards and punishments is beyond the scope of this article, and beyond what can be expected from a lone author. As noted at the beginning of this article, solving the replication crisis requires a full-throated discussion with wide participation across the field. It is a group effort. And we are only just beginning.

Despite points of disagreement, I believe Vize et al. and I are genuinely aligned in our goals: to solve the replication crisis and create a robust and cumulative psychological science.^3^ I hope our exchange can be counted as a constructive part of this ongoing effort.

## Notes

1. Klonsky (2024) argues that turning a scientific tool into a badge, goal, norm, or mandate causes the tool to be misused. Vize et al. (2024) interprets one of these terms, norm, as indicating “a separate concern” about “preregistration itself.” This is an unfortunate misunderstanding. In Klonsky (2024), the terms goal, norm, badge, and mandate are all used in opposition to use of the tool itself (e.g., “As a tool, preregistration can help remedy the file drawer problem . . . In contrast, as a goal and norm for publication, PRM rewards like badges create false confidence”). This use of the term norm comports with Webster’s first definition: “an authoritative standard,” “a principle of right action binding upon the members of a group” (Merriam-Webster, 2024). I regret the confusion. My concern is *not* about preregistration itself. Tools like hypotheses, *p*-values, multi-study designs, and preregistrations have many benefits, just not when they become badges, norms, goals, or mandates.

2. Strictly speaking, sample size does not always refer to number of participants. For example, an experimental case design randomly assigning a treatment to time intervals can yield meaningful inferences about the treatment’s impact on a person, as long as an appropriate number of observations are obtained. In this example, the population is the person’s relevant experiences, and the sample is the observations taken.

3. This article is focused on how to produce, identify, and motivate robust scientific findings. While this enterprise is the foundation of a healthy science, it is not everything required for a healthy science. For example, among many other unaddressed issues (what questions should scientists ask, how can science be made more equitable), this article does not address how scientists convert robust findings into conceptual and theoretical knowledge (Yarkoni, 2022). But first things first: in my mind, we must consistently produce and identify findings we trust before we can discuss how to interpret them.
